# Posterior-Only T11 Vertebral Column Resection for Pediatric Congenital Kyphosis Surgical Correction

**DOI:** 10.3390/medicina60060897

**Published:** 2024-05-29

**Authors:** Pawel Grabala, Negin Fani, Jerzy Gregorczyk, Michal Grabala

**Affiliations:** 1 Department of Pediatric Orthopedic Surgery and Traumatology, Medical University of Bialystok, University Children’s Hospital, Waszyngtona 17, 15-274 Bialystok, Poland; 2 Paley European Institute, Al. Rzeczypospolitej 1, 02-972 Warsaw, Poland; 3 Department of Neurosurgery, Medical University of Bialystok, M. Sklodowskiej-Curie 24A, 15-276 Bialystok, Poland; 4 Medical Faculty, Medical University of Warsaw, 02-091 Warsaw, Poland; neginfani@gmail.com (N.F.); george.gregorczyk@gmail.com (J.G.); 5 2nd Clinical Department of General and Gastroenterogical Surgery, Medical University of Bialystok, ul. M. Skłodowskiej-Curie 24a, 15-276 Bialystok, Poland; michal@grabala.pl

**Keywords:** congenital kyphosis, kyphosis, spinal deformity, vertebral column resection, VCR, vertebrectomy, kyphosis correction

## Abstract

*Background*: Congenital kyphosis is a spinal deformity that arises from the inadequate anterior development or segmentation of the vertebrae in the sagittal plane during the initial embryonic stage. Consequently, this condition triggers atypical spinal growth, leading to the manifestation of deformity. Concurrently, other congenital abnormalities like renal or cardiac defects within the gastrointestinal tract may co-occur with spinal deformities due to their shared formation timeline. In light of the specific characteristics of the deformity, the age range of the patient, deformity sizes, and neurological conditions, surgical intervention emerges as the optimal course of action for such cases. The selection of the appropriate surgical approach is contingent upon the specific characteristics of the anomaly. *Case Presentation*: This investigation illustrates the utilization of a surgical posterior-only strategy for correcting pediatric congenital kyphoscoliosis through the implementation of a vertebral column resection method along with spine reconstruction employing a mesh cage. The individual in question, a 16-year-old female, exhibited symptoms such as a progressive rib hump, shoulder asymmetry, and back discomfort. Non-invasive interventions like bracing proved ineffective, leading to the progression of the spinal curvature. After the surgical procedure, diagnostic imaging displayed a marked enhancement across all three spatial dimensions. After a postoperative physical assessment, it was noted that the patient experienced significant enhancements in shoulder alignment and rib hump prominence, with no discernible neurological or other adverse effects. *Conclusions*: Surgical intervention is considered the optimal approach for addressing such congenital anomalies. Typically, timely surgical intervention leads to favorable results and has the potential to halt the advancement of deformity and curvature enlargement.

## 1. Introduction

Congenital kyphosis (CK) is a spinal deformity that arises from the inadequate anterior formation or segmentation of the vertebrae in the sagittal plane during the early embryonic stage [1,2,3,4]. This condition leads to aberrant spinal growth and the subsequent development of deformity. Additional congenital anomalies, such as renal or cardiac defects within the gastrointestinal tract, may co-occur with the spinal defect due to their simultaneous formation during this developmental period [1,3,4,5]. Congenital kyphosis arises from early embryological anomalies in the segmentation or formation of one or more vertebral bodies. The formation of vertebral bodies takes place during the initial trimester of gestation. Vertebral anomalies emerge as a secondary outcome, stemming from osseous metaplasia of the anterior annulus fibrosus of the intervertebral disk or specific growth defects of the centrum. These anomalies lead to either a deficiency in segmentation or a deficiency in formation, respectively. The presence of congenital spine disorders necessitates an evaluation of other organ systems, given the well-documented correlation between congenital spine disorders and abnormalities in other organ systems during embryological development [1,2,3,4,5,6].

The most prevalent form of kyphotic deformity is typically localized in the thoracic or thoracolumbar region, exhibiting swift progression and a heightened risk of spinal cord compression, potentially resulting in paraplegia if left untreated. Severe spinal curvatures can manifest at various spinal levels, with the thoracolumbar region being predominantly affected in cases of congenital kyphosis. The advancement of the spinal curvature presents variability, yet it predominantly unfolds during the period of spinal growth, exhibiting an accelerated pace between the ages of 1 and 5, as well as from the ages of 10 until skeletal maturity. These stages signify distinct rapid phases of spinal development [3,4,5,6,7].

Progressive deformity can also lead to the emergence of cardiopulmonary dysfunction. The effectiveness of conservative treatment utilizing casts or braces remains uncertain and subject to debate. The clinical manifestation of congenital kyphosis displays variability, with severe forms sometimes evident at birth and milder forms potentially identified during routine assessments of adolescents referred for postural irregularities [4,5,6,7,8]. Taking into account the specific characteristics of the deformity, the age range of the patient, the extent of the deformity, and the neurological status, surgical intervention emerges as the optimal strategy for managing such cases. Recent advancements in surgical methodologies and instruments have established the posterior approach as the preferred technique for conducting posterior vertebral column resection (PVCR) in cases of congenital spinal deformities [5,9,10].

In this study, we performed a surgical procedure to correct pediatric congenital kyphoscoliosis. The procedure involved a posterior-only vertebral column resection via a lateral extracavitary approach at T11 and an anterior spinal fusion for the T10–T12 vertebrae with additionally inserted an anterior structural titanium cage in the VCR site. The procedure was performed on a 16-year-old female who presented with a progressive rib hump, shoulder asymmetry, and back pain.

## 2. Case Presentation

**Figure 1:**
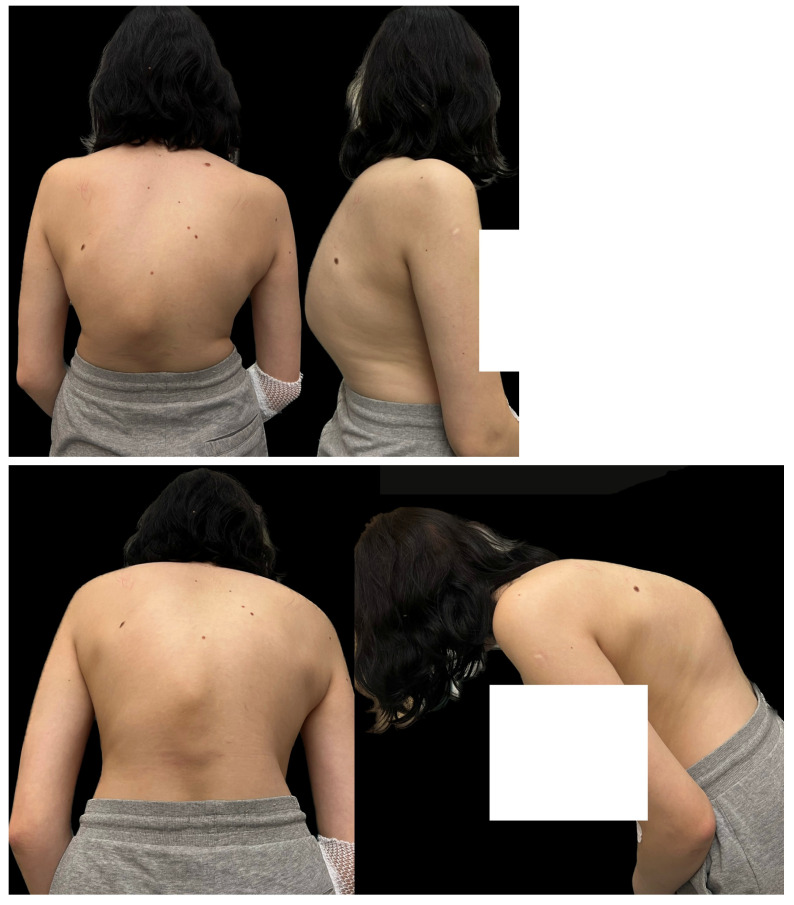
Clinical pictures of the 16-year-old female before surgical treatment. The girl presented with severe congenital kyphosis with a thoraco-lumbar progressed hump.

A 16-year-old female patient came to our clinic because of a progressive spinal deformity. She was diagnosed with a congenital spinal deformity at 9 years of age. Conservative measures such as bracing were used but were unsuccessful, and her curve progressed over time. Additionally, at 9 years of age, an MRI confirmed the absence of intraspinal pathology and congenital thoracolumbar kyphoscoliosis. During the examination in our clinic, she presented with a progressive rib hump and shoulder asymmetry in the Adams forward bend test. She also had a severe thoracolumbar hump, underlying back discomfort and pain when sitting and at night, and a Risser score of 3/4 (Figure 1).

**Figure 2:**
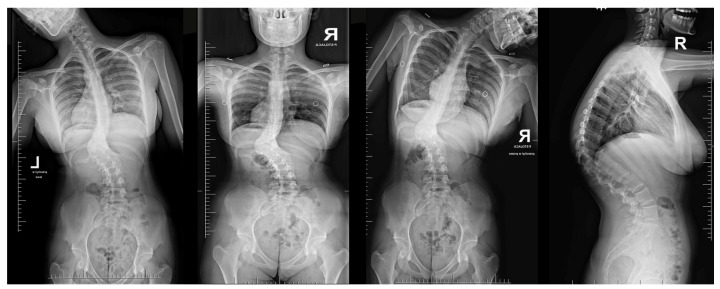
Standard standing AP and lateral X-rays and side-bending films of the 16-year-old female before surgical treatment. These X-rays showed severe and stiff congenital kyphosis.

Neurological examinations showed full strength (5/5) in all major muscle groups of BUE/BLE, sensation intact throughout, normal reflexes (including abdominal), and pain on palpation in the thoracic spine above and below the gibbous. The neuro-imaging findings indicated a Cobb angle of 63° in the main thoracolumbar curve. The flexibility of the curve was measured at 63° when bending, decreasing to 48°. Additionally, there was a thoracic kyphosis of 4° between T2 and T5 and 32° between T5 and T12, with a severe focal thoracic kyphosis of 108° (Figure 2).

**Figure 3:**
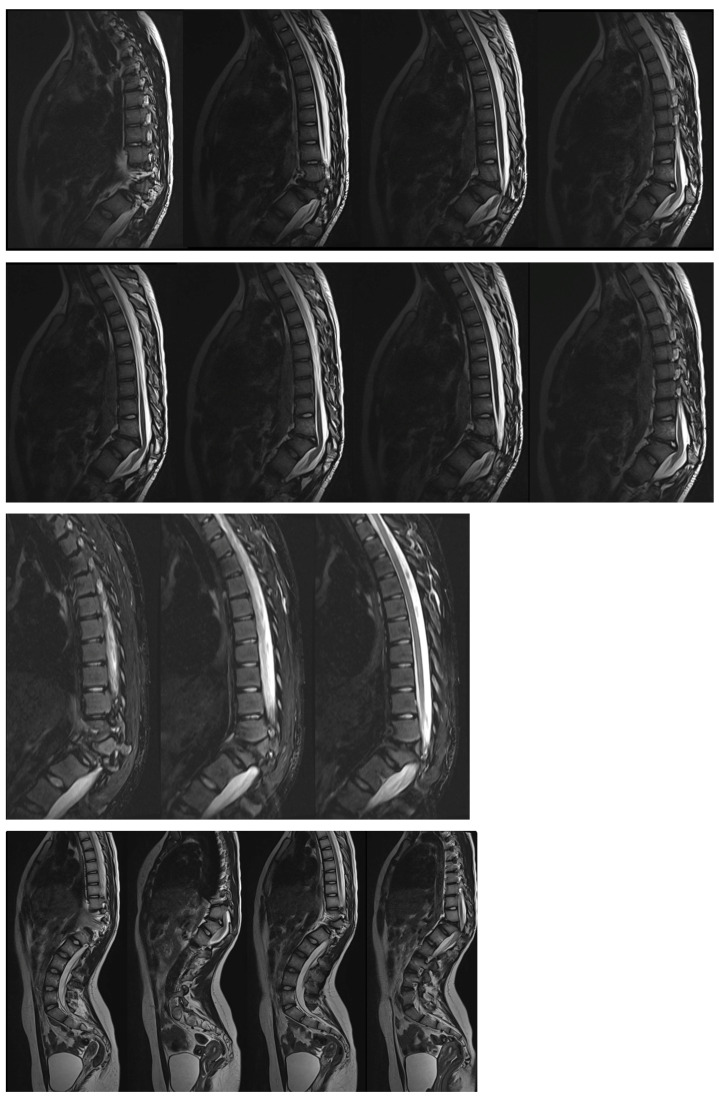
MRI scans showed spinal cord compression in the 16-year-old female before surgical treatment.

The obtained MRI of the thoracic and lumbar spine showed compression of the spinal cord at T10–T12 (Figure 3).

**Figure 4:**
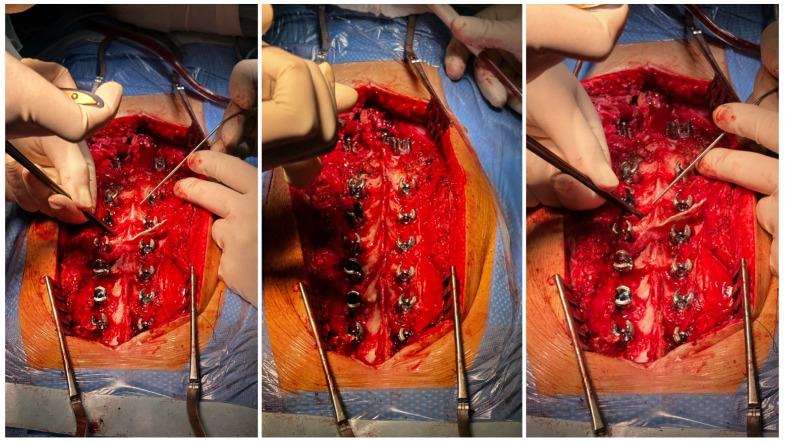
Pedicle screw placement using a free-hand technique under neuromonitoring control from T5 to L3; transverse process hook placed bilaterally at T4 to prevent pullout, and PJK, peri-apical Ponte’s osteotomies (at T9–L1).

**Figure 5:**
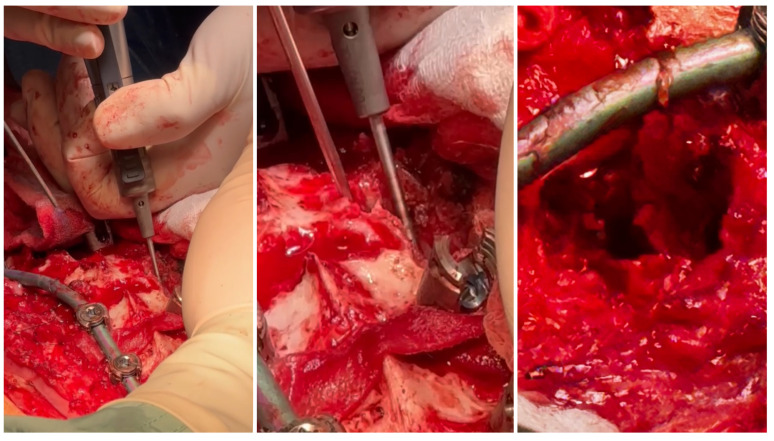
Temporary rod placed, P-VCR at T11 performing power burr, preparing for placement of a titanium mesh cage for anterior column reconstruction.

**Figure 6:**
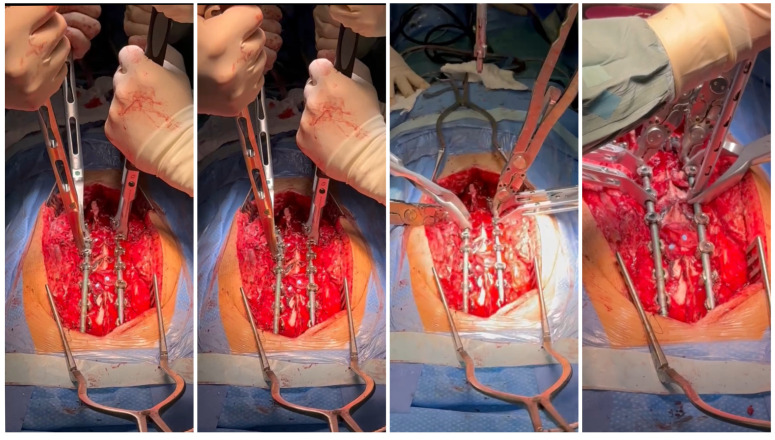
The deformity correction via compression and in situ techniques, and temporary rods being bent and replaced with contoured rods to achieve adequate sagittal and coronal balance.

The patient required surgical treatment. We considered several alternatives for the management of this severe case: continued observation (controversial for congenital cases), continued bracing/casting (controversial for congenital cases), in situ fusion and spinal cord decompression, an anterior/posterior approach, decompression, spinal fusion, lateral retropleural/retroperitoneal corpectomy, and posterior spinal fusion. We analyzed the indications, contraindications, and risks of potential surgical treatment using various techniques. Due to the extensive experience of our center in the comprehensive treatment of severe and neglected congenital spinal deformities, a one-stage treatment using a posterior approach was planned—a posterior-only vertebral column resection via a lateral extracavitary approach at T11 and anterior spinal fusion for the T10–T12 vertebrae with the addition of an anterior structural titanium cage in the VCR site and posterior spinal fusion from the T4 to the L3 levels. The rationale for the procedure was the failure of conservative management; a progressive increase in curve magnitudes over time; progressive mechanical back pain upon sitting, standing, and lying; sagittal imbalance; an inability to maintain a balanced, upright posture for prolonged periods; and, finally, a high risk of neurological deficits and paraplegia. However, like any surgical procedure, the one we planned was burdened with potential complications and the risk of failure. The risks of the procedure include neurovascular injury/spinal fluid leak, neurological deficits, implant prominence, post-surgical junctional kyphosis, adding-on, pull-out of screws/rod fractures/pseudarthrosis, hardware malposition, infections, and revision surgery. The benefits of the procedure are as follows: reconstitution of thoracolumbar junction alignment, improved coronal and sagittal alignment of the spine, stabilization of spine deformity progression, curve correction, global sagittal balance, balancing of shoulder asymmetry, decreased back pain, potential reduction in the risk of paraplegia, and improvement in health-related quality of life [11]. We performed a one-stage posterior surgery, positioning the patient in a prone position on an open Jackson table using neuromonitoring (SSEP, MEP), C-arm, and surgical loupes [12,13]. The most important technical steps for the surgical procedure were: pedicle screw placement using a free-hand technique under neuromonitoring control (6.5 and 60.0 multiaxial screws from T5 to L3; transverse process hook placed bilaterally at T4 to prevent pullout and PJK), peri-apical Ponte’s osteotomies (at T9–L1) as shown in Figure 4, temporary rod placement, P-VCR at T11 using a titanium mesh cage for anterior column reconstruction as presented in Figure 5, deformity correction via compression and in situ techniques, and temporary rods being bent and replaced with contoured rods to achieve adequate sagittal and coronal balance [14] as shown in Figure 6.

**Figure 7:**
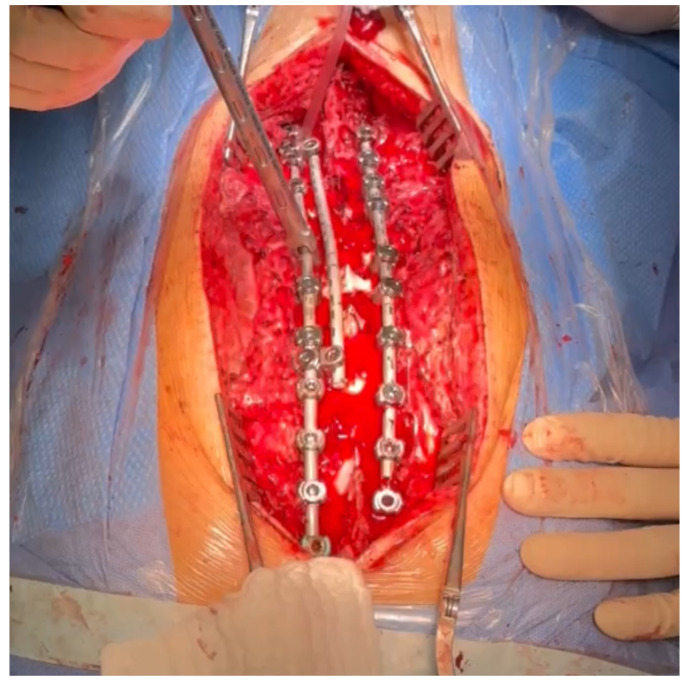
Final correction performed with three 6.0 Co-chr rods.

The procedure involves the installation of 6.0 Co-chr rods, followed by the adjustment of their position and the subsequent addition of a third 6.0 Co-chr rod [15,16] as presented in Figure 7.

We have attached our video surgical technique as Supplementary Materials: Video S1: Posterior-Only T11 Vertebral Column Resection for Pediatric Congenital Kyphosis Surgical Correction.

**Figure 8:**
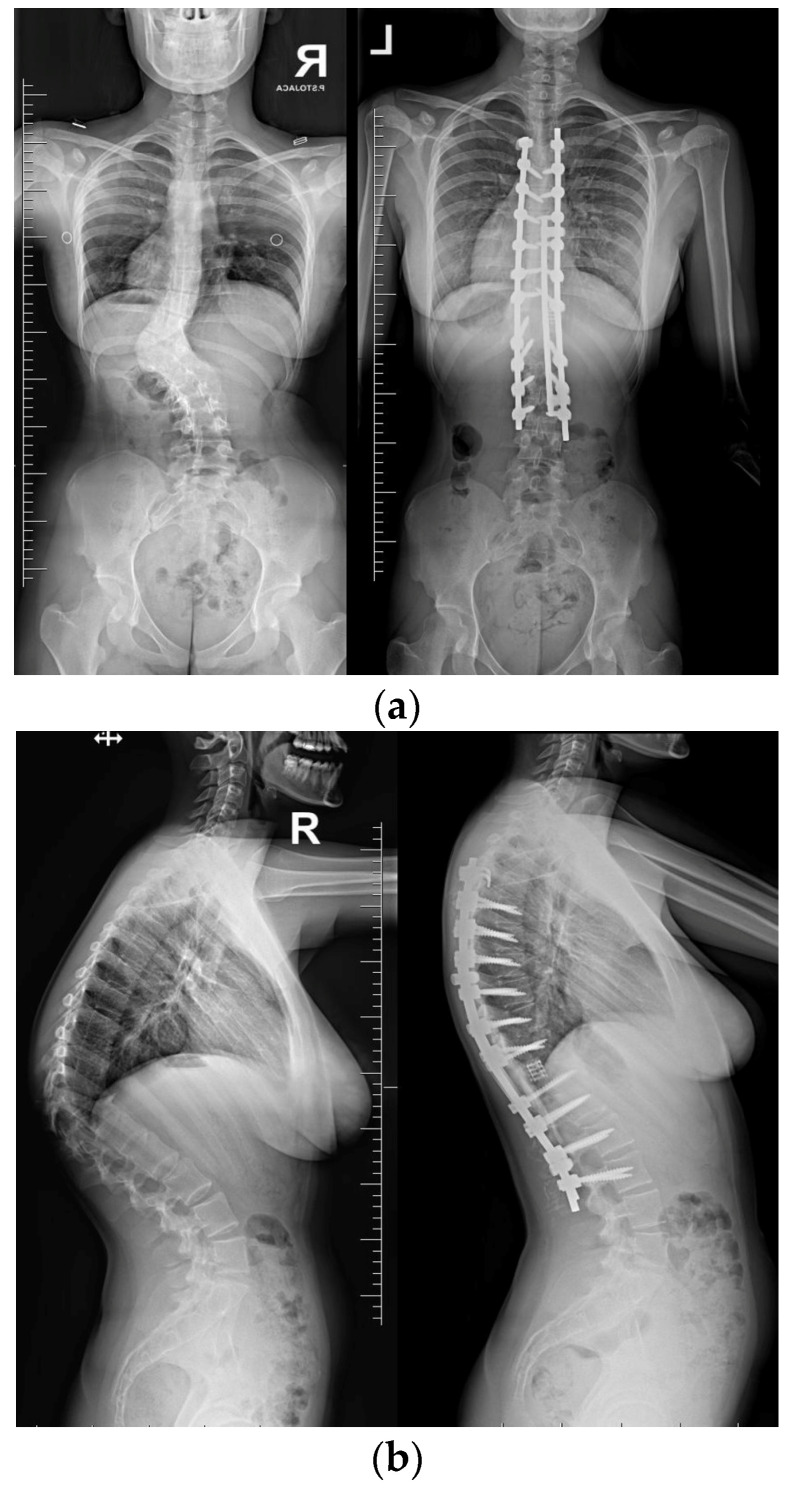
Standard standing AP (**a**) and lateral (**b**) X-rays of the 16-year-old female after undergoing surgical treatment. These X-rays were performed just after surgery.

Postoperative imaging showed significant improvement in both three-dimensional planes (Figure 8).

**Figure 9:**
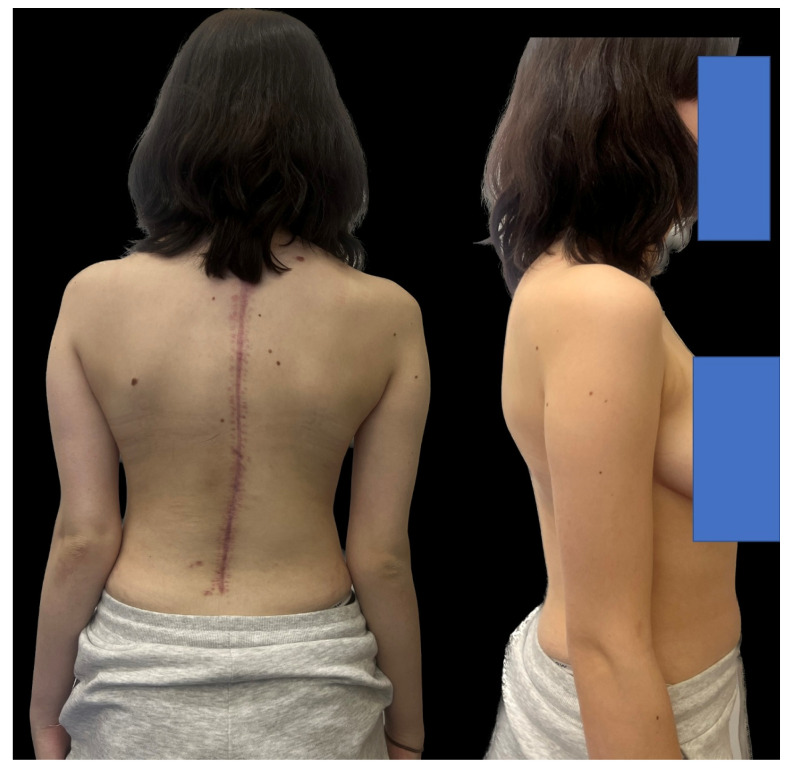
Clinical pictures of the 16-year-old female after surgical treatment.

**Figure 10:**
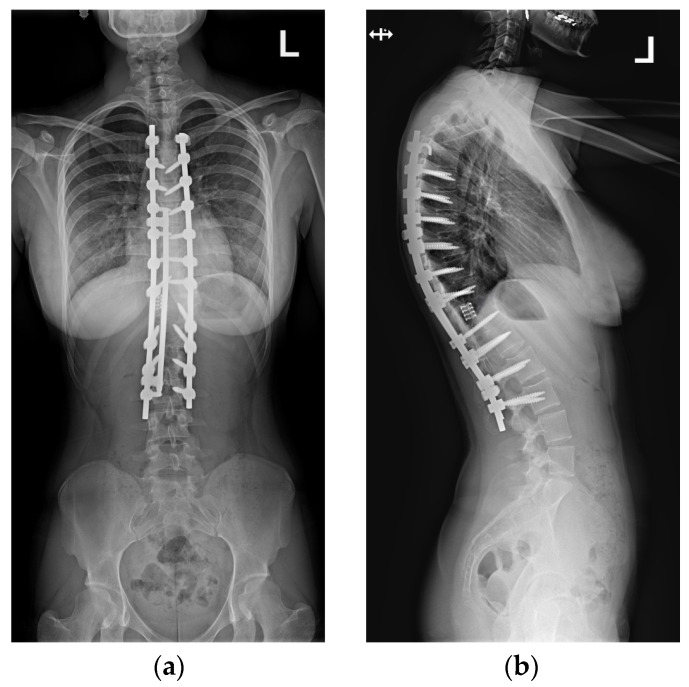
Standard standing AP (**a**) and lateral (**b**) X-rays of the 16-year-old female after undergoing surgical treatment at 2 years of follow-up.

**Figure 11:**
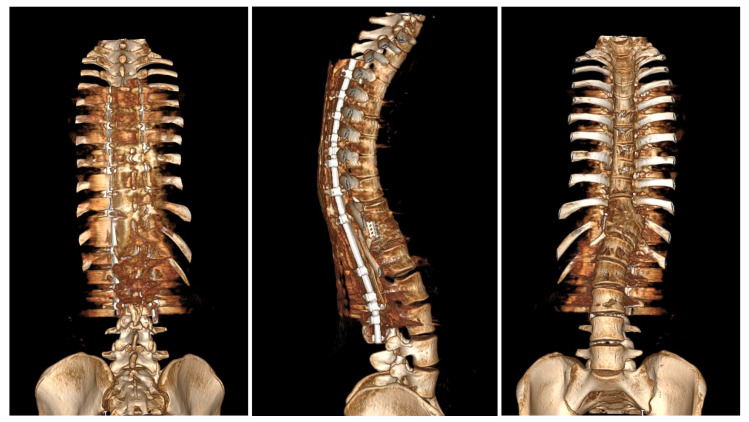
3D-computer tomography reconstruction of the whole spine of the 16-year-old female after undergoing surgical treatment at 2 years of follow-up. The pictures show spondylodesis of the posterior and anterior columns.

Upon conducting a postoperative physical examination, it was observed that the patient’s shoulder symmetry and rib hump had improved significantly without any neurological or other complications. Figure 9 shows a significant improvement in the patient’s posture, sagittal balance (focal thoracic kyphosis from 108° to 43°), and coronal balance (from 63° to 25°). The patient was discharged 5 days after surgery; no brace was ordered. Figure 10 and Figure 11 show the X-rays and 3D-CT of the patient at the final follow-up.

## 3. Discussion

Congenital kyphosis can be categorized into three distinct classifications: anterior spinal column formation defect (type 1), segmentation failure (type 2), and a combination of formation and segmentation failure (type 3). The most prevalent form, Type 1, exhibits a kyphotic deformity primarily situated in the thoracic or thoracolumbar region. This type is characterized by a swift progression and a heightened risk of spinal cord compression, potentially leading to paraplegia if left untreated [4,5,10]. Instances of progressive deformity may also give rise to cardiopulmonary dysfunction. Type 2, on the other hand, poses a lower risk of neurological decline and typically progresses gradually without causing extreme deformities. The cranial lumbar region, or thoracolumbar junction region, is frequently affected by this type. The extent of the deformity and the number of segments implicated in Type II do not consistently align. Type 3 deformities are predominantly observed in segments T10–L-1 and often exhibit a faster rate of progression compared to Type 2 deformities, regardless of their location along the spinal column [2,3,4].

The treatment approach for congenital kyphosis primarily emphasizes operative measures to address curve advancement, as bracing is not considered effective in managing this condition. The choice of surgical intervention is influenced by various factors, including the patient’s age, the specific type of deformity, the degree of curvature, and the presence or absence of neurological symptoms [3,5]. Fundamental principles guiding surgical procedures include safeguarding neurological function and ensuring the achievement of a stable fusion, which can be achieved through either posterior surgery or a combination of anterior and posterior approaches. Congenital kyphosis should be regarded as a progressive condition where the development of severe deformities and the possibility of neurological impairment are prevalent [1,6,7,8,17]. While the progression of curvature may vary, it typically aligns with spinal growth, notably accelerating between ages 1 and 5 and then from age 10 until skeletal maturity, which represents crucial periods of rapid spinal growth. Timely surgical intervention often yields favorable outcomes by halting deformity progression and curbing curve enlargement. The selection of a surgical procedure is contingent upon the specific characteristics of the anomaly [1,3,5,6,7].

The most current recommendation for congenital kyphosis entails surgical management, specifically posterior fusion, and decompression for deformities ranging from less than 50° to 60°. Research suggests the utilization of anterior decompression, anterior fusion, and posterior instrumented arthrodesis for more severe abnormalities that are linked to cord compression [6,8]. The sudden occurrence of paraplegia subsequent to the surgical correction of kyphoscoliosis is extensively documented in existing literature. The advancement of congenital kyphosis has the potential to result in spinal cord compression, paraplegia, and cardiopulmonary dysfunction [1,6]. However, patients might necessitate surgical intervention due to other concurrent conditions such as a tethered cord, syrinx, and Chiari malformation [1,7,18]. Concurrent anomalies may also involve rib irregularities, absent vertebrae, or hemivertebrae and may be correlated with spinal cord or urinary irregularities. Structural irregularities commonly impact pulmonary function, leading to decreased exercise tolerance and ventilation. The onset of cor pulmonale is linked to an unfavorable prognosis and an elevated risk of mortality [2,8,18].

The posterior column resection approach is compelling for addressing moderate-to-severe deformities indicated by restricted flexibility. Nevertheless, it represents a technically demanding and strenuous procedure with potentially significant complications [8,17]. In a recent retrospective study conducted by Mohsen Karami et al., it was observed that subsequent to posterior column resection for rigid congenital spinal deformities in 23 pediatric patients, the most common late postoperative complications were sagittal plane decompensation, affecting eight patients (34%), and coronal decompensation, seen in two patients (8%). Moreover, Mohsen Karami et al. stated that lumbar or thoracolumbar posterior vertebral resection allows surgeons to correct rigid curves in the pediatric population with minimal risk to nerve roots [19]. However, some studies have indicated that individuals with operated congenital kyphosis or kyphoscoliosis demonstrate one of the highest rates of neurological deficits post-operatively [14,20,21,22]. Additionally, Kim et al. identified five instances of paraplegia and pseudoarthrosis among 26 cases of significant complications following congenital kyphosis surgery [8]. Another retrospective study by Johanna Syvänen et al. revealed that, despite posterior vertebral excision carrying a 44% probability of temporary issues, enhanced pain levels led to a significant improvement in health-related quality of life. Nevertheless, this enhancement in health-related quality of life remained relatively modest when compared to a similar health control group. The lack of current research on congenital kyphosis underscores the necessity for further investigations in this particular domain [23].

A research investigation carried out by Zarzycki et al. analyzed the surgical results of congenital scoliosis in nine patients; deformity stabilization was conducted on six patients, while the remaining three showed progression. Improvement in neurologic status was documented in five patients. Paraplegia was observed in one patient. Optimal clinical outcomes were attained following extensive spinal cord decompression with anterior stabilization and posterior spinal fusion through a combined approach [24]. In a literature review of 36 articles conducted by Pahys et al., it was noted that congenital scoliosis encompasses a broad spectrum of conditions ranging from uncomplicated, stable hemivertebra to intricate, advancing spinal deformities with chest wall irregularities and associated cardiac, renal, and neural axis anomalies [2]. In a study by Zhao et al. [25], eleven patients below the age of 10 underwent two-step osteotomy procedures for complex rigid congenital scoliosis. The spinal flexibility mean was 17.4 and 17.8% before the two surgeries, respectively. The average age at the time of the initial surgery was 6.6 years. The scoliosis mean improved from 67.4° to 23.7° after the initial surgery and was 17.4° at the latest follow-up. None of the patients experienced neurological complications. In a study by Xu et al. [26], the authors assessed surgically treated patients using solely the posterior approach. The group comprised 15 CK patients, and the mean Cobb angle of local kyphosis was corrected from 65.6 to 11.3. The average correction rate was 83.2%. The correction remained stable during follow-up, with no recurrence of deformity or junctional kyphosis. These results mirror those of our surgical intervention. Another study [27] examined twenty-nine patients, and the mean local deformity angle notably improved from 94.9° to 24.0°, with no significant regression during follow-up. There were no instances of intraoperative or neural injury in the perioperative phase. Fixation failure was absent at the last follow-up assessment. In the other study [28], catastrophic surgical complications were present in 22.5% of patients, with seven cases of neurological deficits and two fatalities. A notable correlation was noted between the occurrence of major complications and type III congenital kyphosis. Major complications were reported in 30% of patients. Surgical interventions have demonstrated a significant enhancement in addressing symptomatic congenital kyphosis deformity; nonetheless, operating on symptomatic patients may lead to a heightened risk of complications and mortality.

The critical steps for reducing neurological complications during PVCR involve a systematic approach. Initially, performing wide laminectomies, specifically one level above and one level below the osteotomy site, can help avoid spinal cord impingement during the correction process. The initiation of correction by applying compression through the convex side in cases of kyphoscoliosis holds significant importance. In instances of lordoscoliosis, the correction should commence from the concave side on both sides of the osteotomy, moving alternately proximally and distally to the osteotomy site. It is essential to assess the spinal canal following each correction attempt, involving both compression and in situ contouring. Utilizing mesh cages anteriorly serves the dual purpose of filling any remaining gap and preventing excessive shortening and dural buckling. To prevent dural compression by hematoma in the laminectomy region, placement of an H-shaped strut allograft between intact spinous processes is recommended, with a specific sequence of placement followed by final compression [14,29].

Most surgeons performing corrective procedures for deformities now frequently incorporate posterior instrumentation, even in pediatric patients, due to its positive impact on fusion rates [2,3,9]. Modern implant designs are now accessible, demonstrating both effectiveness and anatomical suitability. The decision to utilize autograft or allograft depends on the surgeon; nevertheless, allograft may be a viable option given the constraints related to the limited iliac crest size in children and their significant osteogenic capacity. Complications such as pseudoarthrosis, progressive deformity, hardware failure, and neurological issues can be mitigated through meticulous patient selection and precise surgical methods. The dimensions of a deformity and the utilization of instrumentation greatly influence the occurrence of pseudarthroses. Incorporating instrumentation into a well-prepared fusion site enhances the likelihood of achieving successful fusion. The advancement of a deformity commonly stems from pseudarthrosis, which arises from inadequate execution of a comprehensive fusion procedure.

Curves measuring below 50 degrees can be managed using posterior instrumentation and fusion techniques. Curves exceeding this threshold necessitate anterior arthrodesis with strut grafting, followed by posterior arthrodesis supported by instrumentation. Patients presenting with neurological deficits or severe curves at risk of neurological compromise necessitate immediate anterior neurologic decompression through a direct anterior approach or costotransversectomy [1,2,3,4,5]. Subsequently, posterior arthrodesis and instrumentation are performed. Individuals diagnosed with an isolated hemivertebra, especially in early childhood, constitute optimal candidates for hemivertebra excision via anterior-posterior or posterior-exclusive surgical approaches [5,10]. Despite its rarity, congenital spinal dislocation mandates stabilization through comprehensive surgery, potentially followed by decompression post-restoration of mechanical stability.

### Limitations

We are cognizant of the limitations inherent in our article. The present study constitutes a case report detailing the management of a patient afflicted with congenital kyphosis, elucidating our surgical methodology. It is acknowledged that the formulation of a comprehensive worldview necessitates more than a singular case study; nevertheless, it is our aspiration that the surgical approach outlined herein will furnish valuable insights to fellow practitioners in the realms of clinical decision-making, operative intervention, and the evolution of their individualized treatment protocols for similar clinical scenarios. Robust investigations featuring larger cohorts are imperative to assess the patient’s protracted postoperative quality of life, osseous consolidation, and the incidence of post-surgical complications.

## 4. Conclusions

A remarkable enhancement in the patient’s alignment, sagittal equilibrium (focal thoracic kyphosis), and frontal equilibrium can be attained. The optimal option for addressing these forms of congenital irregularities is surgical intervention, with prompt surgical management typically yielding superior results and halting the progression of deformity and curvature enlargement. Surgical intervention is advisable for individuals who have not shown improvement with conservative therapy, endure persistent pain, or manifest neurological symptoms. In situations of advancement, the compression of the spinal cord can result in severe complications, such as paraparesis or paraplegia, due to neurological compromise. Surgical complications leading to neurological compromise can arise from the intricate task of reinstating spinal stability in instances of three-dimensional deformities.

## Data Availability

Data are contained within the article and Supplementary Materials.

## Supplementary Materials

The following supporting information can be downloaded at https://www.mdpi.com/article/10.3390/medicina60060897/s1; Video S1: Posterior-Only T11 Vertebral Column Resection for Pediatric Congenital Kyphosis Surgical Correction.
